# Nomograms Forecasting Long-Term Overall and Cancer Specific Survival of Patients With Head and Neck Neuroendocrine Carcinoma

**DOI:** 10.3389/fonc.2021.619599

**Published:** 2021-02-15

**Authors:** Ouying Yan, Wenji Xie, Haibo Teng, Shengnan Fu, Yanzhu Chen, Feng Liu

**Affiliations:** ^1^ The Affiliated Cancer Hospital of Xiangya School of Medicine, Central South University/Hunan Cancer Hospital, Changsha, China; ^2^ Department of Radiation Oncology, Hunan Cancer Hospital and The Affiliated Cancer Hospital of Xiangya School of Medicine, Central South University, Changsha, China; ^3^ Department of Neurosurgery, West China Hospital, Sichuan University, Chengdu, China

**Keywords:** head and neck neuroendocrine carcinoma, nomograms, prediction model, cancer‐specific survival, overall survival, SEER

## Abstract

**Background:**

The purpose of this retrospective analysis was to build and validate nomograms to predict the cancer-specific survival (CSS) and overall survival (OS) of head and neck neuroendocrine carcinoma (HNNEC) patients.

**Methods:**

A total of 493 HNNEC patients were selected from the Surveillance, Epidemiology, and End Results (SEER) database between 2004 and 2015, and 74 HNNEC patients were collected from the Affiliated Cancer Hospital of Xiangya School of Medicine, Central South University/Hunan Cancer Hospital (HCH) between 2008 and 2020. Patients from SEER were randomly assigned into training (N=345) and internal validation (N=148) groups, and the independent data group (N=74) from HCH was used for external validation. Independent prognostic factors were collected using an input method in a Cox regression model, and they were then included in nomograms to predict 3‐, 5‐, and 10‐year CSS and OS rates of HNNEC patients. Finally, we evaluated the internal and external validity of the nomograms using the consistency index, while assessing their prediction accuracy using calibration curves. A receiver operating curve (ROC) was also used to measure the performance of the survival models.

**Results:**

The 3-, 5-, and 10-year nomograms of this analysis demonstrated that M classification had the largest influence on CSS and OS of HNNEC, followed by the AJCC stage, N stage, age at diagnosis, sex/gender, radiation therapy, and marital status. The training validation C-indexes for the CSS and OS models were 0.739 and 0.713, respectively. Those for the internal validation group were 0.726 and 0.703, respectively, and for the external validation group were 0.765 and 0.709, respectively. The area under the ROC curve (AUC) of 3-, 5-, and 10-year CSS and OS models were 0.81, 0.82, 0.82, and 0.78, 0.81, and 0.82, respectively. The C-indexes were all higher than 0.7, indicating the high accuracy ability of our model’s survival prediction.

**Conclusions:**

In this study, prognosis nomograms in HNNEC patients were constructed to predict CSS and OS for the first time. Clinicians can identify patients’ survival risk better and help patients understand their survival prognosis for the next 3, 5, and 10 years more clearly by using these nomograms.

## Introduction

Neuroendocrine carcinomas are rare malignancies of the head and neck. HNNEC is rare, accounting for approximately <5% of all head and neck cancers. Most patients present with advanced disease at the time of diagnosis. This may be due to the previous lack of clear pathologic diagnostic criteria. In the previous literature, typical carcinoid and atypical carcinoid neuroendocrine carcinomas were exceedingly rare in HNNECs, perhaps because they had been classified under non-descriptive categories, such as “neuroendocrine carcinoma, not otherwise specified (NOS)”. These tumors often look similar to other sites’ neuroendocrine tumors, and are thus particularly difficult to distinguish (1). Early studies showed that NECs had a varied histopathologic spectrum, and the scope of nomenclature which was used to describe them was very wide but both confusing and ambiguous (2, 3), and the classification did not stress the use of the names “carcinoid” and “atypical carcinoid” as diagnosis categories. This problem with neuroendocrine carcinoma of the head and neck persisted until the World Health Organization (WHO) in 2017 classified NEC into three sub-categories: well-differentiated NEC (typical carcinoid), moderately differentiated NEC (atypical carcinoid), and poorly differentiated NEC (4). The poorly differentiated NEC was further divided into large cell NEC and small-cell NEC (5, 6). Since then, the classification of HNNEC has had a uniform standard. It has been reported that poorly differentiated NEC has an extremely poor prognosis, and the 5-year disease-specific survival was reported to be 19.3% for small-cell NEC and 15.3% for large-cell NEC.

In the early years, the literature on head and neck NEC was limited to case reports (7–15) and small retrospective case series (5, 16–29). In recent years, some systematic reviews (30–39) and meta-analyses (40, 41) have emerged. Research has found that the most common head and neck neuroendocrine carcinomas (NECs) are laryngeal neuroendocrine carcinoma (LNEC), sinonasal neuroendocrine carcinoma (SNEC), and salivary gland neuroendocrine carcinoma (42).

The most common clinical manifestation of salivary gland NEC is a progressively expanding neck mass arising from the parotid or submandibular glands (39). The main symptoms of SNEC are nasal obstruction, epistaxis, nasal drainage, and facial pain (43), and those of LNEC are throat discomfort, dysphagia, hoarseness, and a neck mass (44, 45).

HNNEC is an exceedingly rare entity that is highly malignant, aggressively invasive, and has a high relapse rate and a poor prognosis. There is a paucity of data on the prognostic factors influencing survival in HNNECs; therefore, it often presents diagnostic uncertainty and therapeutic challenges, and patients may be initially misdiagnosed. As a result, larger sample sizes are needed to confirm the relationship between clinical factors and prognosis, and further research is required to explore the risk factors associated with HNNECs.

There was also a need to construct a prognostic prediction model to accurately predict the survival of this cancer. For this reason, we used the Surveillance, Epidemiology, and End Results (SEER) database to construct a prognostic model related to CSS and OS in patients with HNNECs to adjust clinical practice and improve patient survival.

## Methods

### Data Source

The SEER database is supported by the National Cancer Institute as an authoritative source of information on population-based cancer incidence and survival, and it is considered the gold standard for cancer registry worldwide (46). This is an openly available, validated, and deidentified database. Thus, our research of SEER was not required for informed consent and was accepted by the Ethical Committee and Institutional Review Board of The Affiliated Cancer Hospital of Xiangya School of Medicine, Central South University.

The SEER 22 database covers data from the years 2004 to 2015 from 22 cancer registries throughout the United States. Cases collected were done based on the International Classification of Diseases for Oncology, Third Edition (ICD-O-3) topography, and histology/behavior codes. Using the SEER database, 70 sites in the head and neck location were collected and analyzed. The following codes were included in this study. Oral cavity, oropharynx, and hypopharynx including malignant neoplasms from C01.9 (base of tongue) to C06.9 (mouth, NOS), C09.0 (tonsillar fossa) to C09.9 (tonsil, NOS), C10.0 (vallecula) to C10.9 (oropharynx NOS), C12.9 (pyriform sinus) to C13.9 (hypopharynx NOS), C14.0 (pharynx, NOS), and C14.8 (overlapping lesion of the lip, oral cavity, and pharynx). Nasopharynx included C11.0 (superior wall of the nasopharynx) to C11.9 (nasopharynx, NOS). Nasal cavity and sinuses included C30.0 (nasal cavity) to C31.9 (accessory sinus, NOS). Larynx included C32.0 (glottis) to C32.9 (larynx, NOS). Subsequently, we filtered the cases based on ICD-O-3 histology/behavior codes covering large-cell neuroendocrine carcinoma (8013/3), small-cell neuroendocrine carcinoma, NOS (8041/3), typical carcinoid (8240/3), neuroendocrine carcinoma, NOS (8246/3), and atypical carcinoid tumor (8249/3).

### Patients and Clinicopathologic Factors

Patients contained in this study had to satisfy the eligibility criteria: (a) The patients with HNNECs were enrolled between 2004 and 2015, (b) HNNEC was their first or only primary diagnosis, (c) The diagnosis was verified by histological examination, and (d) Complete and integrated follow-up data of patients could be available. The exclusion criteria included the following: (a) Patients were younger than 20, (b) Information about the tumor stage and the follow-up data was lacking,and(c) Death certificate and autopsy cases were also excluded. Finally, 493 eligible HNNEC’s patients were chosen from the SEER database. A random split‐sample approach was applied to separate the total eligible patients into a training cohort (n=345) and internal validation cohort (n=148) at a split ratio of 7:3, using the “caret” package in R version 3.6.3. At the same time, 74 patients with pathological diagnosis of HNNEC from our medical center between 2008 and 2020 were included in the external validation cohort. This retrospective validation was approved by the Ethics Committee of our hospital. (The ethical approval number: Medical Ethics Committee of Hunan Cancer Hospital 2021 Scientific Research Express No. 1).

**Figure 1:**
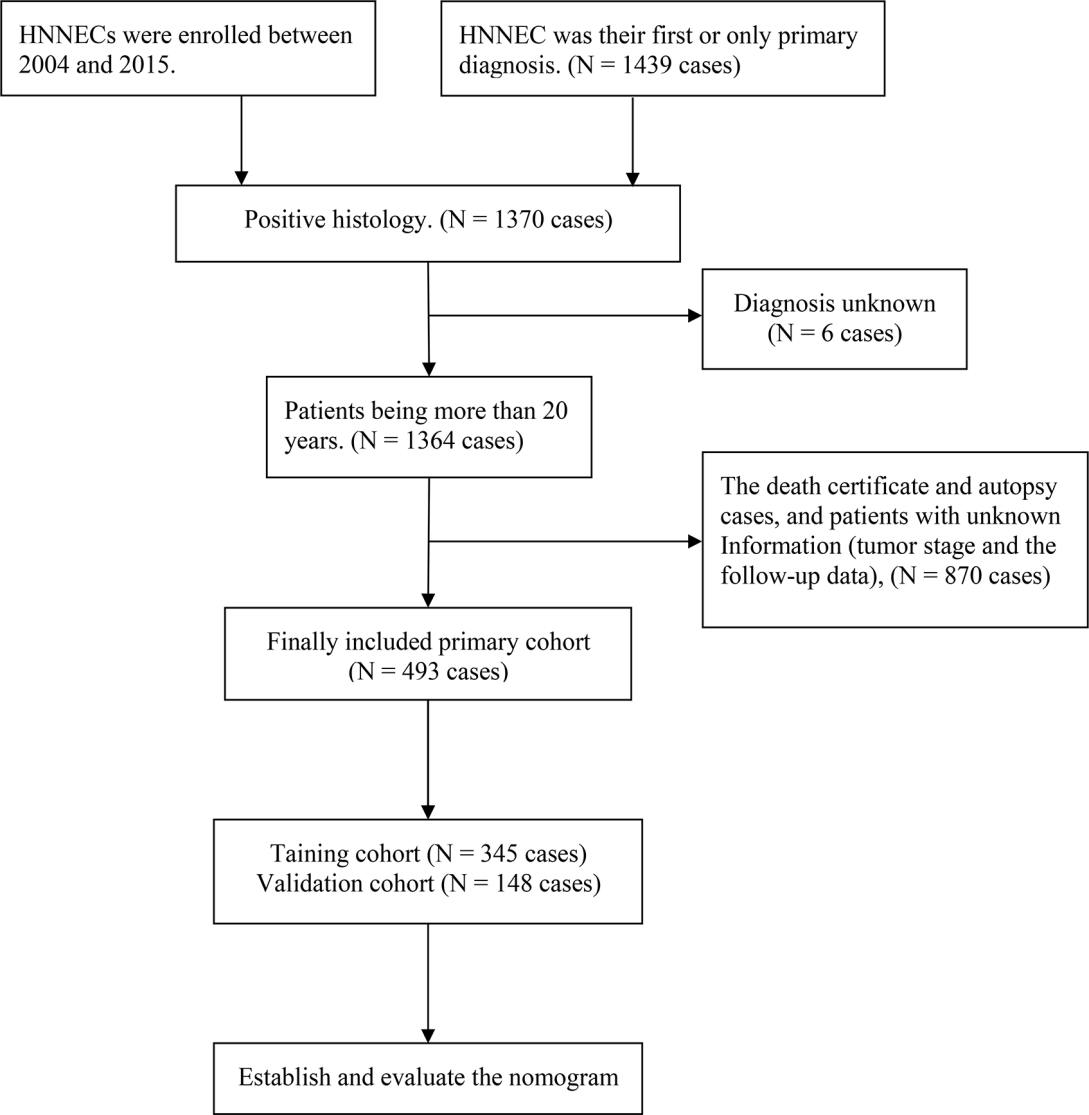
The flow chart of Surveillance, Epidemiology, and End Results data selection.

The detailed screening process is presented in **Figure 1**. We analyzed patients’ demographic characteristics such as age, gender, race, year of diagnosis, and marital status, and also assessed clinical pathology features of the primary site, histological grade, AJCC stage, surgery status, radiotherapy status, and chemotherapy status. It is worth noting that the criteria for grade classification in this analysis is according to previous versions before the new World Health Organization (WHO) 2017 classification. The TNM staging system was confirmed according to the AJCC (American Joint Committee on Cancer) Cancer Staging Manual (sixth edition for diagnosis before 2010 and seventh edition for diagnosis in 2010 or later).

### Statistical Analysis

We carried out the analysis of the above-mentioned factors descriptively. Overall survival (OS) measured the time from the date of diagnosis until the date of death from any cause or last follow-up, and the cancer special survival (CSS) was estimated from the interval between the HNNEC diagnosis until the cancer-associated death or the last follow-up. Then the Kaplan–Meier method was applied to plot survival curves, using the log-rank test to analyze the differences in survival. Both univariate and multivariate Cox regression modeling was employed to evaluate the significant prognostic factors of CSS and OS. By calculating the hazard ratios (HR) and 95% confidence intervals (95% CI), the relationship between the risk factors and survival could be assessed. All analyses were completed by the statistical software SPSS 22.0 and R version 3.6.3. The results of P<0.05 were selected as statistically significant.

### Construction and Validation of the Nomograms

Using the independent prognostic factors identified in the multivariate analysis, nomogram models of CSS and OS were constructed. The calibration plot and concordance index (C-index) was conducted to evaluate the performance of the CSS and OS nomograms in the training, internal validation, and external validation cohorts successively. The C-index ranges from 0 to 1.0, with 1.0 expressing a perfect predictor and 0.5 indicating a completely random prediction. In addition, we plotted the ROC curves and computed the areas under ROC (AUC) to evaluate the model forecasting capability.

## Results

### Patient Clinicopathological Data

**Table 1** shows the demographic and clinical characteristics of the SEER research population. Total patients in our study have a mean age of 62 and a median age of 63. Over half (n =327, 66.3%) of the patients were male. A total of 227 (46.0%) patients were diagnosed at the age of 60-79 years. Most patients 430 (87.2%) were white. The common sites of HNNEC were salivary gland 137 (27.8%), nasal cavity and sinuses 124 (25.2%), and larynx 117 (23.7%). Most of the patients in the overall cohort were grade III/IV (60.8%), AJCC stage IV (62.5%), with a distant metastasis (79.3%), and married (55.6%). Concerning treatment options, more than two-thirds of the patients had received chemotherapy (67.8%), with approximately half of the patients receiving surgery (50.5%) and radiotherapy (41.0%). Of these patients, 316 (64%) died and the median survival was 24.0 ± 3.3 months, with the median follow-up time of 19 months (range 1-154 months).

**Table 1:** Baseline demographics and clinical characteristics of the patients with HNNEC.

Variable	All patients(n=493)	Training cohort(n=345)	Validation cohort(n=148)	p-value
**Age(years) n (%)**				0.577
20-39	36(7.2)	26(7.5)	10(6.7)	
40-59	163(33.3)	110(31.9)	53(35.8)	
60-79	227(46.0)	160(46.4)	67(45.2)	
≥80	67(13.5)	49(14.2)	18(12.1)	
**Gender n (%)**				0.809
Female	166(33.7)	115(33.3)	51(34.4)	
Male	327(66.3)	230(66.7)	97(65.5)	
**Race n (%)**				0.756
White	430(87.2)	301(87.3)	129(87.1)	
Black	38(7.7)	28(8.1)	10(6.7)	
Other ethnicity	25(5.1)	16(4.6)	9(6.0)	
**Year of Diagnosis n (%)**				0.382
2004-2009	70(14.2)	164(47.5)	64(43.2)	
2010-2015	423(85.8)	181(52.5)	84(56.7)	
**Location n (%)**				0.848
Nasopharynx	33(6.7)	20(5.8)	13(8.7)	
Nasal cavity and sinuses	124(25.2)	91(26.4)	33(22.2)	
Larynx	117(23.7)	79(22.9)	38(25.6)	
Salivary Gland	137(27.8)	99(28.7)	38(25.6)	
Oral cavity,oropharynx, and hypopharynx	82(16.6)	56(16.2)	26(17.5)	
**Histology n (%)**				0.796
Large cell neuroendocrine carcinoma	33(6.7)	22(6.4)	11(7.4)	
Small cell carcinoma, NOS	231(46.9)	161(46.7)	70(47.2)	
Carcinoid tumor, NOS	3(0.6)	3(0.9)	0(0)	
Neuroendocrine carcinoma, NOS	222(45.0)	157(45.5)	65(43.9)	
Atypical carcinoid tumor	4(0.8)	2(0.5)	2(1.3)	
**Grade n (%)**				0.219
Grade I/II	41(8.3)	28(8.1)	13(8.7)	
Grade III/IV	300(60.8)	204(59.1)	96(64.8)	
Unknown	152(30.8)	113(32.7)	39(26.3)	
**AJCC stage n (%)**				0.980
I	62(12.6)	43(12.5)	19(12.8)	
II	45(9.1)	32(9.3)	13(8.7)	
III	78(15.8)	55(15.9)	23(15.5)	
IV	308(62.5)	215(62.3)	93(62.8)	
**T classification n (%)**				0.824
T1	110(22.3)	81(23.5)	29(19.5)	
T2	130(26.4)	92(26.7)	38(25.6)	
T3	88(17.8)	52(15.0)	36(24.3)	
T4	139(28.2)	100(29.0)	39(26.3)	
Tx	26(5.3)	20(5.8)	6(4.0)	
**N classification n (%)**				0.574
N0	209(42.4)	146(42.3)	63(42.5)	
N1	83(16.8)	57(16.5)	26(17.5)	
N2	172(34.9)	118(34.2)	54(36.4)	
N3	20(4.1)	17(5.0)	3(2.0)	
Nx	9(1.8)	7(2.0)	2(1.3)	
**M classification n (%)**				0.427
M0	391(79.3)	276(80.0)	115(77.7)	
M1	96(19.5)	66(19.1)	30(20.2)	
Mx	6(1.2)	3(0.9)	3(2.0)	
**Radiotherapy n (%)**				0.181
Yes	202(41.0)	148(42.9)	54(36.4)	
No	291(59.0)	197(57.1)	94(63.5)	
**Surgery n (%)**				0.035
Yes	249(50.5)	185(53.6)	64(43.2)	
NO	244(49.5)	160(46.4)	84(56.7)	
**Chemotherapy n (%)**				0.314
Yes	334(67.8)	229(66.4)	105(70.9)	
NO	159(32.2)	116(33.6)	43(29.0)	
**Marital status n (%)**				0.359
Married	274(55.6)	199(57.6)	75(50.6)	
Divorced	56(11.4)	33(9.6)	23(15.5)	
Single (never married)	85(17.2)	61(17.7)	24(16.2)	
Widowed	78(15.8)	52(15.1)	26(17.5)	
3-years OS (%)	44.3	43.2	–	–
5-years OS (%)	36.5	35.8	–	–
10-years OS (%)	26.2	24.9	–	–
3-years CSS (%)	50.9	50.3	–	–
5-years CSS (%)	43.0	42.5	–	–
10-years CSS (%)	35.0	34.3	–	–

**Table S1** presents the demographic and clinical characteristics of Hunan Cancer Hospital. The median patient age was 52 years (20–75 years) in the HCH cohort. Consistent with the SEER cohort, HNNEC tended to occur more frequently among men (n =58, 78.3%). The common sites of HNNEC originated in the nasal cavity, sinuses (n =26, 35.1%), and nasopharynx (n =24, 32.5%). In our medical center cohort, most of the patients had AJCC IV stage (n =37, 50%), with high grade (n =48, 65%), and no distant metastasis (n =67, 90.5%). The majority of patients received radiotherapy (n =49, 66.2%) and chemotherapy (n =51, 68.9%). At the cutoff date of December 12, 2020, of the HCH patients, 44 (59.4%) died and the median survival was 26.0 ± 4.6 months, with the median follow-up time of 20 months (range 2-144 months).

### Survival Statistics

**Figure 2:**
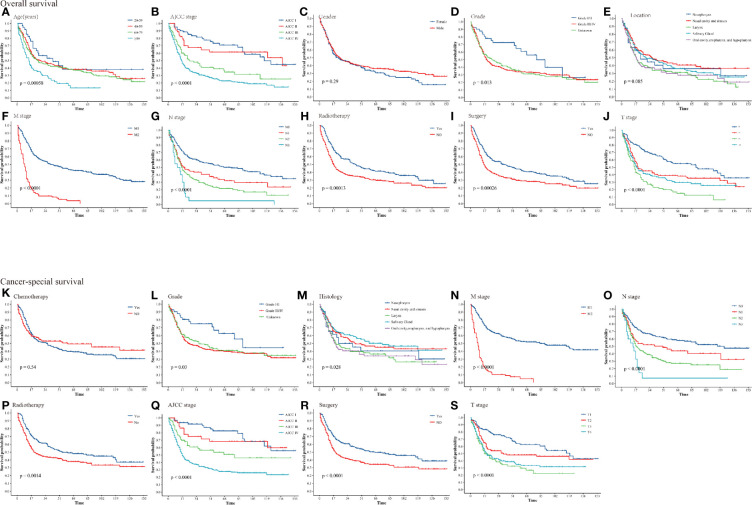
Kaplan–Meier curves for patients with HNNECs by different variates. **(A)** Age, **(B)** AJCC stage, **(C)** Gender, **(D)** Grade, **(E)** Location, **(F)** M stage, **(G)** N stage, **(H)** Radiotherapy, **(I)** Surgery, **(J)** T stage for OS; **(K)** Chemotherapy, **(L)** Grade, **(M)** Histology, **(N)** M stage, **(O)** N stage, **(P)** Radiotherapy, **(Q)** AJCC stage, **(R)** Surgery, **(S)** T stage for CSS.

In SEER, we obtained believable data on CSS and OS for 493 HNNECs patients. In total, 316(64.0%) patients in the overall cohort died by the time of the last follow-up. Among these patients, 261(52.9%) died due to HNNEC and 55(11.2%) died because of causes other than HNNEC. The OS rates of the overall cohort at 3, 5, and 10 years were 44.3%, 36.5%, and 26.2%, respectively. The CSS rates of the overall cohort at 3, 5, and 10 years were 50.9%, 43.0%, and 35.0%, respectively. Cumulative incidence curves by age at diagnosis, race, gender, stage, and treatment were presented in **Figure 2**. For the training cohort, the overall 5-year survival rate of grades (I/II) and grades III/IV were 23.6% and 35.5%, respectively. The 5-year cancer special survival rates of them were 38.4% and 51.6%.

### Independent Predictors for Patients

Univariate and multivariate analyses of characteristics associated with the CSS and OS were presented in **Table 2**. For the training cohort, univariate analysis manifested that the age, gender, AJCC stage, T stage, N stage, M stage, surgery, and radiation therapy were associated with OS significantly. (P<0.05); The tumor location, AJCC stage, T stage, N stage, M stage, surgery, radiation therapy, and marital status were correlated with CSS significantly. (P<0.05).

**Table 2:** Univariate analysis of OS and CSS in the training cohort.

Variables	Univariate analysis (OS)	Univariate analysis (CSS)
	P-value	P-value
Age(years)	**0.025**	0.774
Gender	**0.041**	0.092
Race	0.767	0.769
Year of Diagnosis	0.697	0.680
Location	0.060	**0.009**
Histology	0.110	0.545
Grade	**0.012**	**0.032**
AJCC stage	**＜0.001**	**＜0.001**
T classification	**＜0.001**	**0.001**
N classification	**＜0.001**	**＜0.001**
M classification	**＜0.001**	**＜0.001**
Radiotherapy	**＜0.001**	**0.001**
Surgery	**＜0.001**	**＜0.001**
Chemotherapy	0.749	0.218
Marital status	0.088	**0.035**

We made the p value less than 0.05 bold, which means that the variable has significant meaning.

Multivariate analysis was conducted to identify the independent predictors of HNNCE. Multivariate Cox proportional hazard regression models of OS showed that age≥80 years (HR = 2.072 vs 20-39 years, P=0.023), being male (HR =0.694 vs female, P=0.01), AJCC stage III (HR = 2.415 vs AJCC stage I, P=0.033), N2 (HR =1.741 vs N0, P=0.08), N3 (HR =2.915 vs N0, P=0.01), M1 (HR =3.300 vs M0, P<0.001), and not receiving radiotherapy (HR = 1.473 vs radiotherapy, P = 0.038) were risk factors associated with OS. Multivariate Cox analysis of CSS indicated that AJCC stage III (HR = 3.115 vs AJCC stage I, P=0.024), AJCC stage IV (HR = 3.526 vs AJCC stage I, P=0.009), N3 (HR =2.338 vs N0, P=0.012), M1 (HR =3.629 vs M0, P<0.001), and being widowed (HR = 1.674 vs married, P=0.036) were risk factors connected with CSS. (**Table 3**, **4**).

**Table 3:** Selected variables by OS multivariate Cox regression analysis (training cohort).

Variables	Multivariate analysis		
	HR	95% CI	P-value
**Age(years)**			
20-39	Reference		
40-59	0.900	0.508-1.592	0.716
60-79	0.987	0.565-1.726	0.963
≥80	2.072	1.106-3.883	**0.023**
**Gender**			
Female	Reference		
Male	0.692	0.523-0.916	**0.010**
**Grade**			
Grade I/II	Reference		
Grade III/IV	1.284	0.676-2.441	0.445
Unknown	1.256	0.648-2.436	0.499
**AJCC stage**			
I	Reference		
II	1.501	0.615-3.664	0.372
III	2.323	1.025-5.263	**0.043**
IV	1.955	0.895-4.269	0.092
**T classification**			
T1	Reference		
T2	0.916	0.568-1.476	0.718
T3	1.294	0.760-2.203	0.342
T4	1.202	0.733-1.970	0.467
Tx	0.785	0.394-1.564	0.491
**N classification**			
N0	Reference		
N1	1.191	0.759-1.868	0.448
N2	1.721	1.138-2.603	**0.010**
N3	2.827	1.510-5.292	**0.001**
Nx	0.960	0.318-2.900	0.942
**M classification**			
M0	Reference		
M1	3.300	2.303-4.728	**＜0.001**
Mx	2.456	0.499-12.093	0.269
**Radiotherapy**			
Yes	Reference		
No	1.500	1.036-2.171	**0.032**
**Surgery**			
Yes	Reference		
NO	1.103	0.769-1.582	0.595

We made the p value less than 0.05 bold, which means that the variable has significant meaning.

**Table 4:** Selected variables by CSS multivariate Cox regression analysis (training cohort).

Variables	Multivariate analysis		
	HR	95% CI	P-value
**Location**			
Nasopharynx	Reference		
Nasal cavity and sinuses	0.503	0.242-1.045	0.066
Larynx	1.260	0.653-2.430	0.490
Salivary Gland	0.516	0.251-1.059	0.071
Oral cavity, oropharynx, and hypopharynx	0.819	0.413-1.624	0.568
**Grade**			
GradeI/II	Reference		
GradeIII/IV	2.040	0.984-4.231	0.055
Unknown	1.763	0.827-3.757	0.142
**AJCC stage**			
I	Reference		
II	2.302	0.896-7.574	0.127
III	3.115	1.245-8.857	**0.024**
IV	3.526	1.379-9.017	**0.009**
**T classification**			
T1	Reference		
T2	0.777	0.462-1.307	0.341
T3	1.366	0.768-2.427	0.288
T4	1.423	0.818-2.473	0.212
Tx	1.102	0.547-2.219	0.786
**N classification**			
N0	Reference		
N1	1.181	0.716-1.950	0.515
N2	1.373	0.863-2.182	0.181
N3	2.064	1.053-4.047	**0.035**
Nx	0.838	0.278-2.522	0.753
**M classification**			
M0	Reference		
M1	3.629	2.471-5.330	**＜0.001**
Mx	2.222	0.453-10.906	0.325
**Radiotherapy**			
Yes	Reference		
No	0.649	0.425-0.992	**0.046**
**Surgery**			
Yes	Reference		
NO	0.682	0.430-1.083	0.105
**Marital status**			
Married	Reference		
Divorced	1.136	0.671-1.924	0.636
Single (never married)	1.378	0.925-2.052	0.115
Widowed	1.674	1.078-2.598	**0.022**

We made the p value less than 0.05 bold, which means that the variable has significant meaning.

### Prognostic Nomogram for OS and CSS

**Figure 3:**
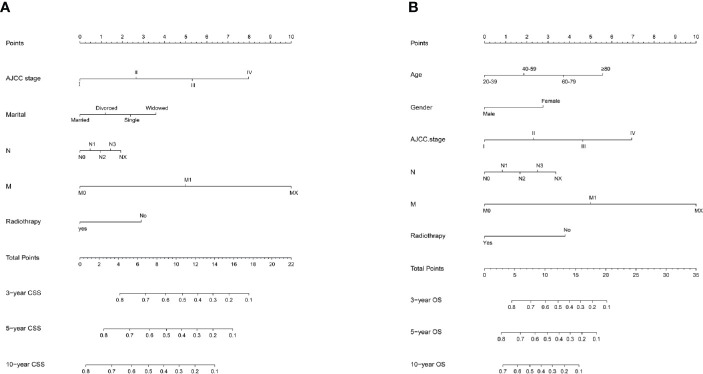
Prediction model nomogram used to predict the cancer-specific survival rate **(A)** and overall survival rate **(B)**.

**Figure 4:**
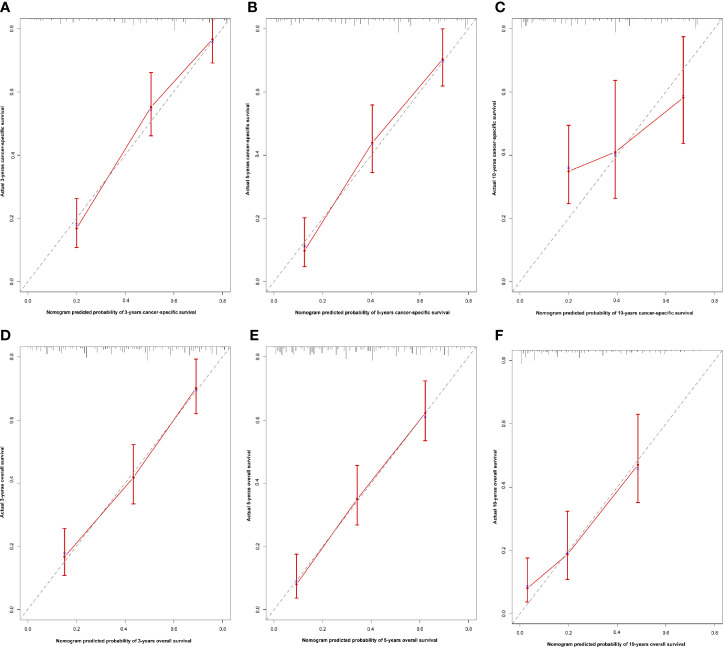
Calibration nomogram for 3- year, 5- year, and 10- year OS **(A–C)** and 3- year, 5- year, and 10- year CSS **(D–F)**. X–axis represents the nomogram predicted survival, and Y–axis expresses the actual survival. The gray dashed line demonstrates the exact match between predicted and actual values of survival. The red vertical line means ±95% confidence intervals.

**Figure 5:**
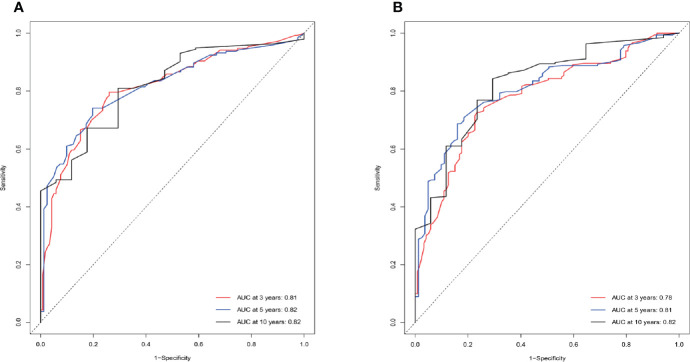
ROC curves of the nomogram predicting 3-year, 5-year, and 10-year CSS **(A)** and OS **(B)** of HNNECs in the training cohort. The ability of the prediction model to be evaluated by the AUC.

**Figure 6:**
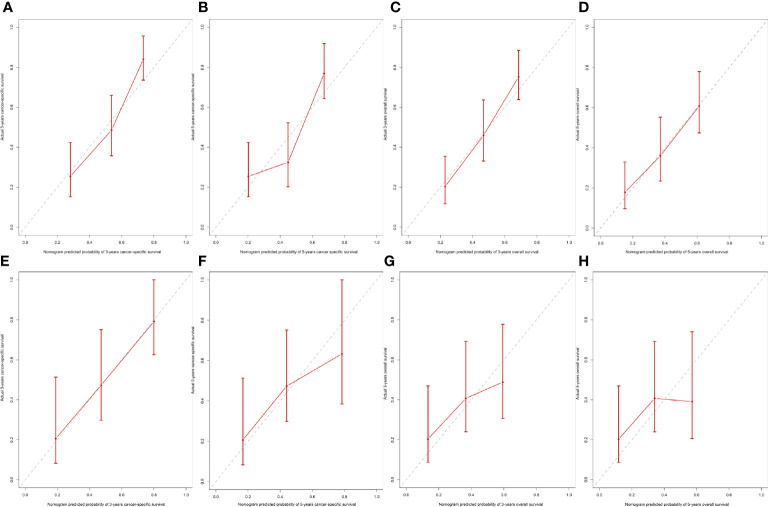
Internal and external calibration of the nomogram. Internal calibration for **(A)** 3- and **(B)** 5-year CSS, and **(C)** 3- and **(D)** 5- OS nomogram calibration curves; External calibration for **(E)** 3- and **(F)** 5-year CSS, and **(G)** 3- and **(H)** 5- OS nomogram calibration curves.

**Figure 7:**
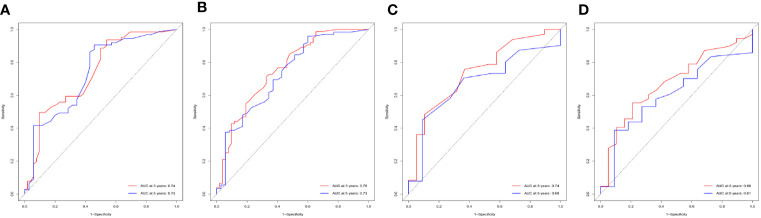
Performance of nomogram by ROC curves. ROC curves for CSS **(A)** and OS **(B)** of patients in the internal validation cohort, and for CSS **(C)** and OS **(D)** in the external validation cohort.

Age, gender, AJCC stage, N stage, M stage, radiation therapy, and marital status were the independent prognostic factors integrated to establish prognostic nomograms for evaluating the 3-, 5-, and 10-year OS and CSS of HNNEC patients (**Figure 3**). Nomogram provides every variable of a fraction on a logarithmic scale. Thus, by summing each variable fraction to get the total points at the bottom scale of the nomogram, we can predict the 3-, 5-, and 10-year OS and CSS of HNNEC patients. In this study, the nomograms were not only conducted using internal validation but also external. The C-index of OS nomogram was 0.713, 0.703, and 0.709 for training, internal validation, and external validation groups, respectively. The C-index of CSS nomogram was 0.739, 0.726, and 0.765 for training, internal validation, and external validation groups, respectively. The value of the C-index was all higher than 0.7; these indicated that there was good concordance between the predicted probability and the actual probability in the models. The values for area under ROC curve (AUC) of 3-, 5-, and 10-year for OS were 0.78, 0.81, and 0.82, respectively, and for CSS were 0.81, 0.82, 0.82, respectively (**Figure 5**). The internal validation cohort’s AUC values for the CSS and OS were 0.74, 0.73, and 0.76, 0.73. The external validation cohort’s AUC values for the CSS and OS were 0.74, 0.68, and 0.68, 0.61 (**Figure 7**). The calibration curve also manifested a well-calibrated of nomogram model (**Figures 4**, **6**).

## Discussion

A total of 493 patients diagnosed with HNNEC were collected in the SEER database between 2004 and 2015. First, we developed and validated nomogram models for HNNEC based on clinical features and treatment modalities. Additionally, the calibration curves and the ROC curve showed a high prediction accuracy of the proposed nomograms. When external validation was performed on the Independent medical center cohort, the C-indexes for OS or CSS were all higher than 0.7. These results proved the high accuracy and excellent discrimination ability of our models’ survival prediction.

Existing analysis of prognosis in HNNEC is usually achieved by comparison with the OS or CSS. In recent years, some studies using the SEER database have been published. Patel et al. reported on 201 SNEC cases in 2015. They compared DSS (CSS) between patients with SNECs. Their results indicated that some adverse prognostic factors influence the survival of SNEC, including tumor locations such as ethmoid and maxillary involvement, treatment plan such as radiotherapy without surgical resection, and AJCC stages III and IV (44). Ghosh et al. performed an analysis of 257 LNEC patients who were diagnosed between 1973 and 2011. Equivalent prognosis results were found in the LNEC group. Unfortunately, perhaps due to the lack of independent prognostic factors, the above studies only analyzed the characteristics and survival outcomes of cancer, without building nomogram prognostic models. Based on this, the main purpose of our study is to establish and validate prognostic nomogram models for HNNECs to forecast the 3-, 5-, and 10-years CSS and OS.

In our study, an OS nomogram was developed based on six independent prognostic factors, including age, sex/gender, AJCC stage, N stage, M stage, and radiotherapy. A CSS nomogram was developed based on four independent prognostic factors: AJCC stage, N stage, M stage, and marital status. Every variable corresponds to a fraction on the logarithmic scale of each nomogram. The fractions for all variables were summed to obtain the total score, and a vertical line could be drawn straight from the total‐points scale to evaluate the chance of surviving for 3, 5, and 10 years.

Our two nomograms included several prognostic variables that are used in routine clinical practice. Some research studies have found an inclination for the nomogram score to increase with the M classification (44, 47). This trend matches the results of our study. Our multivariable analyses indicated that M1 stage was an independent risk factor for both CSS and OS, and it had the highest fraction in both nomogram models. In other words, patients with distant metastases had a dismal prognosis.

Retrospective research studies based on the SEER database found that survival for grade III or IV (20.5%) LNEC was significantly lower than that for grade I or II (60.2%). Low histological grades (I/II) were observed to have better 5-years CSS than grades III/IV, which is consistent with our results (47). Although this is only a laryngeal neuroendocrine carcinoma dataset, it remains persuasive in some sense because it is one of the most common HNNEC tumors. Similar results were also observed in another study that investigated sinonasal neuroendocrine carcinoma (44). In addition, the multivariable analyses showed that the AJCC III stage was an independent risk factor for OS, and the AJCC III stage and AJCC IV stage are independent risk factors for CSS. A higher AJCC stage also has a higher score in both nomograms, and the same is true for the N classification. These clearly indicated that higher AJCC stage and N classification are indicators of poor prognosis. A similar result was seen in a meta-analysis that included 436 reported cases, which may be due to a high proclivity for the late stages to metastasize distantly and recur.

Age and sex/gender are the secondary factors for OS but not CSS. This finding is reasonable because CSS pays more attention to cancer itself. In our analysis, older age indicated a worse prognosis. Previous literature has reported that the average age at the time of diagnosis was between 55 and 56 years for SNEC (17, 44). In addition, a meta-analysis that included 701 cases by van der Laan et al. reported that the average age at the time of diagnosis was between 60 and 63 years for LNEC (40). This is in agreement with the above studies. The average age at the time of diagnosis of 493 HNNEC patients in this analysis was 62.4 years, with a standard deviation of 14.6 years. This suggests that HNNEC is most frequently diagnosed in the age range of 48 to 77 years. This paper has shown that the majority of patients were white (83.2%), which was also in accordance with prior studies (47).

Furthermore, we also found a male predilection in our study, with a male to female ratio of 1.97:1. The reason for this sexual discrepancy might be that a higher proportion of men smoke (40, 47). However, whether gender was a risk factor for HNNEC patients had not been confirmed previously, but this study found that being female was an independent risk factor for overall survival (HR = 0.692, p= 0.01).

Moreover, we found that being married was a protective variable of CSS. It influenced the survival of patients with HNNEC. A report indicated that unmarried patients, including the widowed and divorced, were at higher risk of tumor metastasis, inadequate treatment, and death from cancer (48). The reason for this possibility is that married patients had more active desire and better adherence to treatment. Patients of HNNEC may have one or more comorbidities, and the economic burden also increases rapidly with comorbidities. Single, divorced, and widowed patients have more psychological vulnerability and economic burdens, while married patients may often be accompanied by their spouses, have more emotional and financial support, seek medical attention more frequently, and have a lower burden of comorbidities than unmarried patients. In addition, a review proved that being married positively affects the probability of early diagnosis of cancer. Accordingly, an unmarried person was more likely to suffer from this disease and had a relatively shorter life expectancy (49). In our study, such patients included those who were single, divorced, or widowed (HR = 1.674, p= 0.022).

Apparently, undergoing surgery could improve the survival rate significantly according to univariate analyses. However, this was not significant in multivariate analysis. In our two nomogram models, we found that those who underwent postoperative radiotherapy could obtain a lower score than those who didn’t. In other words, patients with postoperative radiotherapy showed better survival rates. This was confirmed in both univariate and multivariate analyses. This phenomenon was also reported in a meta-analysis that found that surgery plus radiotherapy with or without chemotherapy was associated with a lower risk of death (50).

Our survival curve also indicated that both surgery and surgery combined with radiotherapy were significantly associated with improved outcomes in HNNEC patients (5-year CSS of 53.2% versus 29.1%, p < 0.001, and 51.8% versus 34.8%, p = 0.0014, respectively). However, chemotherapy did not provide greater outcomes in these patients, and the application of chemotherapy did not improve the overall survival of these patients (5-year CSS of 37.7% versus 58.2%, p= 0.54).

We found that the most common locations for HNNEC were the salivary gland, nasal sinuses, and larynx, which is in agreement with previous reports. While univariate analysis reached statistical significance, multivariate Cox regression analysis showed that location may not be an independent prognostic factor. Thus, it was not included in our nomogram.

An earlier study by Likhacheva et al. (2011) found that pathologic classification may not be a crucial factor in the clinical therapy of sinonasal NEC (5). However, Tom P et al. (2015) suggested that the treatment outcome of laryngeal neuroendocrine carcinoma was strongly dependent on histological subtype (40) and Tom P et al. (2016) proposed that histological diagnosis was the most influential factor affecting the efficacy and survival of Head and Neck NECs (50). The reason for these seemingly contradictory results is unclear. This may be due to the absence of a widely accepted histological grading system. For the existing grade classification, there is a lack of intelligibly described criteria and practical clinical significance. Indeed, the possibility that the report provided inadequate data to confirm the histological nature of the tumor cannot be ruled out. Univariate analysis of our research showed that grade was significantly associated with OS and CSS. The probable explanation may be that small cells, large cells, and NEC NOS are inclined to be higher grades. Small-cell NEC has been reported in 50% of patients with positive lymph nodes at the initial diagnosis, and 90% of all patients had distant metastasis (51, 52). However, no significant difference was recognized by multivariate analysis in our study.

As far as we know, other nomograms have been built to predict the survival of head and neck cancer previously, including those for nasopharyngeal carcinoma, tongue squamous cell cancer, salivary gland cancer, and adenoid cystic cancer, among others. However, our analysis is the first to design a nomogram for HNNEC, and this nomogram could help physicians make clinical decisions. For example, there are two HNNEC patients in AJCC stage III: one is a 31-year-old married man who received radiotherapy after surgery, while the other patient is a 77-year-old widowed woman who underwent surgery only, without radiation therapy. According to the AJCC staging system, these two patients would yield a duplicate prognosis. However, applying our nomograms could produce a different result: the 3-, 5-, and 10-year OS rates of the former patient were 80%, 74%, and 62%, respectively, and those of the latter were 32%, 22%, and 12%, respectively. The corresponding CSS rates were 76%, 70%, 60%, and 42%, 31%, 19%, respectively.

## Limitations

There were also some potential limitations to our study. Although the strength of the SEER database is its relatively greater ability to provide more data for studies of rare cancers, this is a retrospective study and lacks specific treatment details. For example, SEER data did not include the specific chemotherapy regimen and could not distinguish between sequential and concurrent chemoradiation. In addition, details such as local control rate, cigarette use, alcohol consumption, and toxic reactions are not available for research. We used the data of our medical center to externally validate the nomogram model, due to the rare incidence in our single hospital and the extremely small number of HNNEC patients, a multi-centered, large sample study is still required to further validation. In the future, we look forward to prospective studies to test nomograms to compensate for these limitations.

## Conclusion

In conclusion, we have established an easy-to-use visual nomogram with several clinical and pathological factors to predict the survival risk of HNNECs. A fraction corresponding to HNNEC patients’ prognostic factors would be acquired on the nomogram point scale. Adding these scores to the total on the bottom scale could predict the 3-, 5-, and 10-year OS and CSS of HNNEC patients.

## Data Availability Statement

The datasets presented in this study can be found in online repositories: https://seer.cancer.gov/data-software/documentation/seerstat/.

## Ethics Statement

The studies involving human participants were reviewed and approved by Medical Ethics Committee of Hunan Cancer Hospital. Written informed consent for participation was not required for this study in accordance with the national legislation and the institutional requirements.

## Author Contributions

OYY and FL contributed to conception and design, methodology and investigation, and study supervision. OYY and WJX contributed to data collection, data analysis and interpretation and writing and review. HBT contributed to data analysis and interpretation and the software. SNF and YZC contributed to the validation. All authors contributed to the article and approved the submitted version.

## Funding

This study was supported by grants from Hunan Provincial Science and Technology Department (Hunan Provincial Natural Science Foundation of China, No. 2016JJ6088), Beijing Hope Run Special Fund of Cancer Foundation of China (No. LC2016W05 and LC2016W06), and Health Commission of Hunan Province (No. B2016048 and C2017044). The funders had no role in study design, data collection and analysis, decision to publish, or preparation of the manuscript.

## Conflict of Interest

The authors declare that the research was conducted in the absence of any commercial or financial relationships that could be construed as a potential conflict of interest.

## Supplementary Material

Supplementary Table 1 Baseline demographics and clinical characteristics of the patients with HNNEC in our medical center.
