# Supplementary material for: Nomograms Forecasting Long-Term Overall and Cancer Specific Survival of Patients With Head and Neck Neuroendocrine Carcinoma
Source: Front Oncol. 2021 Feb 15;11:619599. doi: 10.3389/fonc.2021.619599 (PMC7917297; doi:10.3389/fonc.2021.619599)
Supplement: Supplementary Table 1 — Baseline demographics and clinical characteristics of the patients with HNNEC in our medical center. [file Table_1.docx]

**SUPPLEMENTARY TABLE 1** Baseline demographics and clinical characteristics of the HCH patients with HNNEC.

| Variable | All patients  (n=74) | Variable | All patients  (n=74) |
| --- | --- | --- | --- |
| **Age(years) n (%)** |  | **T classification n (%)** |  |
| 20-39 | 14(19) | T1 | 6(8.1) |
| 40-59 | 36(48.6) | T2 | 23(31.1) |
| 60-79 | 24(32.4) | T3 | 20(27.1) |
| ≥80 | 0(0) | T4 | 24(32.4) |
| **Gender n (%)** |  | Tx | 1(1.3) |
| Female | 16(21.6) | **N classification n (%)** |  |
| Male | 58(78.3) | N0 | 27(36.4) |
| **Race n (%)** |  | N1 | 6(8.1) |
| White | 0(0) | N2 | 25(33.7) |
| Black | 0(0) | N3 | 15(20.2) |
| Other ethnicity | 74(74) | Nx | 1(1.3) |
| **Year of Diagnosis n (%)** |  | **M classification n (%)** |  |
| 2008-2009 | 2(2.7) | M0 | 67(90.5) |
| 2010-2020 | 72(97.2) | M1 | 6(8.2) |
| **Location n (%)** |  | Mx | 1(1.3) |
| Nasopharynx | 24(32.5) | **Radiotherapy n (%)** |  |
| Nasal cavity and sinuses | 26(35.1) | Yes | 49(66.2) |
| Larynx | 9(12.2) | No | 25(33.8) |
| Salivary Gland | 6(8.1) | **Surgery n (%)** |  |
| Oral cavity,oropharynx, and hypopharynx | 9(12.1) | Yes | 30(40.5) |
| **Histology n (%)** |  | NO | 44(59.4) |
| Large cell neuroendocrine carcinoma | 1(1.4) | **Chemotherapy n (%)** |  |
| Small cell carcinoma, NOS | 32(43.2) | Yes | 51(68.9) |
| Carcinoid tumor, NOS | 1(1.4) | NO | 23(31.1) |
| Neuroendocrine carcinoma, NOS | 38(51.3) | **Marital status n (%)** |  |
| Atypical carcinoid tumor | 2(2.7) | Married | 69(93.2) |
| **Grade n (%)** |  | Divorced | 2(2.7) |
| Grade I/II | 5(6.7) | Single (never married) | 2(2.7) |
| Grade III/IV | 48(65) | Widowed | 1(1.3) |
| Unknown | 21(28.3) | 3-years OS (%) | 36.5 |
| **AJCC stage n (%)** |  | 5-years OS (%) | 33.7 |
| I | 4(5.4) | 10-years OS (%) | 16.8 |
| II | 13(17.6) | 3-years CSS (%) | 65.3 |
| III | 20(27) | 5-years CSS (%) | 45.5 |
| IV | 37(50) | 10-years CSS (%) | 34.1 |
